# An overview of viral mutagenesis and the impact on pathogenesis of SARS-CoV-2 variants

**DOI:** 10.3389/fimmu.2022.1034444

**Published:** 2022-11-28

**Authors:** Muhammad Zafar Irshad Khan, Adila Nazli, Hawaa Al-furas, Muhammad Imran Asad, Iqra Ajmal, Dildar Khan, Jaffer Shah, Muhammad Asad Farooq, Wenzheng Jiang

**Affiliations:** ^1^ College of Pharmaceutical Sciences, Zhejiang University, Hangzhou, Zhejiang, China; ^2^ Faculty of Biological Sciences, Department of Pharmacy, Quaid-i-Azam University, Islamabad, Pakistan; ^3^ International Cooperative Laboratory of Traditional Chinese Medicine Modernization and Innovative Drug Development, Ministry of Education (MOE) of China, Guangzhou City Key Laboratory of Precision Chemical Drug Development, School of Pharmacy, Jinan University, Guangzhou, China; ^4^ Shanghai Key Laboratory of Regulatory Biology, Institute of Biomedical Sciences, East China Normal University, Shanghai, China; ^5^ Department of Health, New York, NY, United States

**Keywords:** mutation in viruses, virulence, SAR-CoV-2, COVID-19, phylogenomics

## Abstract

Viruses are submicroscopic, obligate intracellular parasites that carry either DNA or RNA as their genome, protected by a capsid. Viruses are genetic entities that propagate by using the metabolic and biosynthetic machinery of their hosts and many of them cause sickness in the host. The ability of viruses to adapt to different hosts and settings mainly relies on their ability to create *de novo* variety in a short interval of time. The size and chemical composition of the viral genome have been recognized as important factors affecting the rate of mutations. Coronavirus disease 2019 (Covid-19) is a novel viral disease that has quickly become one of the world’s leading causes of mortality, making it one of the most serious public health problems in recent decades. The discovery of new medications to cope with Covid-19 is a difficult and time-consuming procedure, as new mutations represent a serious threat to the efficacy of recently developed vaccines. The current article discusses viral mutations and their impact on the pathogenicity of newly developed variants with a special emphasis on Covid-19. The biology of severe acute respiratory syndrome coronavirus 2 (SARS-CoV-2), its mutations, pathogenesis, and treatment strategies are discussed in detail along with the statistical data.

## Graphical Abstract

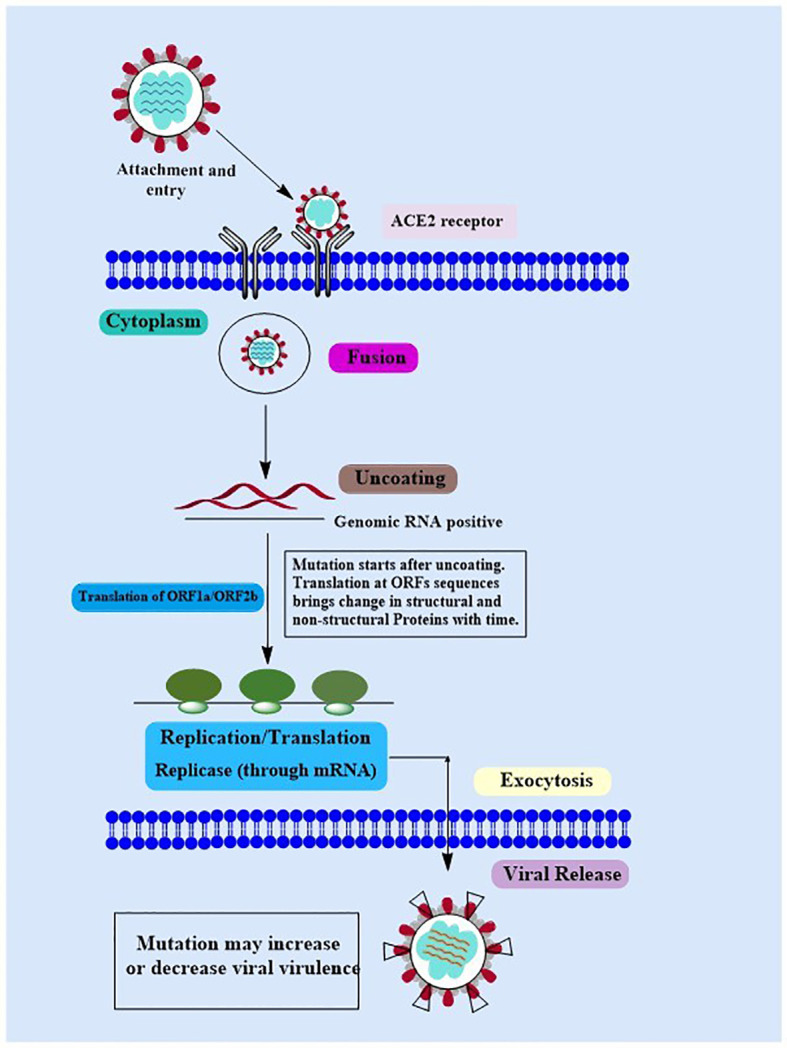

## 1 Introduction

Mutations are reported in many species of living organisms and are mainly considered as a building block of evolution. Mutations cause changes in the living organism which may be beneficial or harmful for the organism itself and other organisms (1). Normally every organism tries to be stable and adapt itself to the surrounding environment to avoid mutation. Various contributing factors compel an organism to adapt accordingly but the majority of the mutations occur due to the biochemical process occurring inside the living organism such as errors in replication, editing, or damage to a nucleic acid. Similarly, mutation rate and adaptation theory are other contributing factors that determine the rate of molecular evolution. The association between evolution and mutation is resolute but it is quite challenging to find out how much they are interdependent. It is because the evolutionary process is directly affected by ecological, selective, and demographical factors (2, 3). Evolution is relatively faster in viruses as compared to other living organisms, thus changes can be easily reported in a timescale of years if matched with the collected isolates of the same virus species (4, 5). The high speed of virus mutation is a constant threat in different magnitudes such as the development of drug resistance, pathogenesis, the success of antiviral treatment, vaccine efficacy, and the likelihood of the emergence of new disease or increasing the virulence of the existing disease (6, 7). Mutations are not usually induced due to replication but can also result from editing or continuous damage to genetic material (nucleic acid). Normally, viruses with an increased rate of mutations can efficiently evade immunity. We have many examples of viruses with high mutation rates causing chronic infections such as hepatitis B virus (HBV), hepatitis C virus (HCV) and human immunodeficiency virus-1 (HIV-1) (8).

## 2 Virulence

Traditionally, one of two methods has generally been used to study viral virulence: theoretically or empirically. Even though both theory and empiricism have produced substantial and comparable insights, however, they can only portray a percentage of pathogenic evolution. Several efforts have been taken so far to overcome this barrier (9, 10). In a range of circumstances, a long-standing evolutionary theory addresses the level of virulence that improves pathogen viability. Different transmission strategies, co-infection rates, selection pressures inside and between hosts are all examples of such scenarios (11). On the other hand, empirical research uses laboratory-based techniques to uncover the virulence determinants (genetic variations that alter virulence) by employing a blend of cell culture, animal models and reverse genetics (12, 13). However, mutations discovered in experimental investigations aren’t evaluated from an evolutionary perspective hence, their significance is largely neglected for basic assumptions of virulence evolution. Additionally, *in vitro* methods may not truly depict the genuine genetic changes hence, little consideration may be given to understand how virulence alteration influence inter-host transmission and animal models usually vary from those infected in the field. Following the appearance of the virus in the host is, arguably, the most intriguing element of virulence evolution (14, 15).

The subject of how virulence will develop is frequently discussed following the advent of a novel virus or the the entry of an old virus with a modified host range. To derive extraordinary insight, investigators must compare pathogenicity in both donor and novel (receiver) host species. Although this may appear to be simple and manageable in certain circumstances, it is actually full of challenges (16). In many cases, such as hepatitis C virus and emerging infectious illnesses like Zika virus (ZIKV), the host species is dubious or uncertain (17). Even when a reservoir species is recognized, there is still a lot of work to be done as nothing is known about its virulence, and there is likely to be a parameter estimation predisposition toward the most virulent circumstances. Better sampling, which is generally inadequate in animals, may also change the identification of host species. For instance, the canine parvovirus (CPV), which initially infected dogs in the late 1970s, was long thought to be the result of a virus that hopped from cats (18). However, subsequent rigorous screening of wild predatory species has revealed that this is incorrect, and the real CPV repository species is unknown. As a result, it’s critical to think about pathological conditions in reservoir hosts in natural settings, especially virulence, which will require more in-depth animal ecological studies. However, less is documented about virulence in host species, statistical information suggests that less virulent viruses are more likely than highly virulent viruses to create transmission cycles in humans (19). This greater probability is assumed to be attributable to the fact that high pathogenicity demands a larger availability of vulnerable hosts during infection’s early phases.

## 3 Mutations rates in RNA and DNA viruses

Among all biological systems, viruses exhibit the most varied rates and patterns of mutation. Similarly, great variations of mutations are observed between RNA and DNA viruses. However several forms of estimating error and distortion impair the credibility of some of these rates (20). Considering these limitations, it may be predicted that viral alteration rates vary between 10^-8^ and 10^-4^ variations per nucleotide per cell infection (s/n/c), with DNA viruses lying between 10^-8^ and 10^-6^, and RNA viruses falling between 10^-6^ and 10^-4^. There are several different processes that might account for these variations. Firstly, a significant proportion of RNA viral polymerases miss a 3′ exonuclease sequencing function, making them more error-prone than DNA virus polymerases (21, 22). Coronaviruses are an exception to this rule, as they encode a complex RNA-dependent RNA polymerase with a 3 exonuclease domain (23). Due to the absence of the 3 exonuclease domain in reverse transcriptases (RTs), retroviruses (viruses with RNA-containing virions and a cellular DNA stage) and para-retroviruses (viruses with DNA-containing virions and a cellular RNA stage) evolve and grow at rates that are comparable to those non-reverse transcribing RNA viruses (24, 25). The difference between DNA viruses and RNA/RT viruses is widely understood from both a genetic and a mechanistic point of view, although distinctions in biological evolution are less evident (26). African swine fever virus (ASFV), tomato yellow leaf curl geminivirus, human parvovirus, beak-and-feather disease circovirus, and canine parvovirus strains have all been found to mutate at comparable rates to RNA viruses (27). This underlines the fact that evolution is impacted by various factors other than mutation rate, and that many DNA viral genetic alterations are undiscovered and may be more frequent than commonly assumed. Although this estimation was preliminary, current findings with human cytomegalovirus has an average genome-wide s/n/c of 2 10^-7^, which is somewhat different from the results assumed for a huge double-strand DNA virus. Given the fact that nearly all DNA and RNA viruses have similar life cycles and face similar experimental conditions, it’s unknown why these two types of genetic alterations have grown so significantly (28).

Mutation rates are predicted to balance various factors such as detrimental impacts of most new mutations, the adaptive effects of a small number, and the costs of mutagenesis management. A random mutation rate of 0.003 per genome per replication has emerged in microbial species that retain their genomes in DNA (20, 29). RNA viruses are said to have substantially higher rates of spontaneous mutation. However, rather than mutation rates themselves, this assumption is based almost entirely on assessments of mutant frequencies and evolution rates. There are several factors, the most crucial of which are population history, selection, replication mechanism, and mutability at each replication phase which are connected to mutation rates in natural and experimental populations. Mutant frequencies can therefore differ greatly from mutation rates. The highest permissible harmful mutation rate cannot be substantially higher than 1 per genome per replication with or without modifying variables like high fecundity, huge populations, and recombination (30, 31).

## 4 Virus phylogenomics

Phylogenetics is a field of molecular epidemiology that presumes information about taxonomy and microorganism evolution (32). It is a potent method that has already been employed in the study of rapidly developing RNA viruses and bacteria using phylogenetic analysis in nosocomial infection (33–35). Phylogenetic analyses of viruses, especially those that include entire genomes, are prevalent and are widely employed to explore a variety of viral evolution-related aspects. A growing number of studies use virus phylogenies to examine the advancement of essential phenotypic traits like pathogenicity. Phylogenomics is a useful tool for understanding virulence evolution since it generates a series of assumptions that may be evaluated using appropriate investigations (9, 10). Phylogenomics can be utilized to test generic virulence evolution theories, using theoretical and empirical methods to examine the virulence of nature. The main component of this method is projecting changes onto phylogenetic trees of viruses collected from reservoirs and new hosts throughout and/or between epidemics. The phylogenetic placement of such alternations that take place either alone or in combination, on shallow or deep nodes (branches) allows researchers to estimate the selection forces acting on virulence mutations, and hence crucial features of virulence evolution. The greater the fitness of a virulence determinant, the more quickly it will propagate across the viral population and the further it will fall on a phylogeny of viruses (i.e., nearer to the root of the tree), also on the branch leading from the reservoir to the new hosts (36). Multiple cross-species transmission events or the same mutation occurring again on deep branches throughout multiple outbreaks are particularly relevant since both parallel and convergent evolution may be signs of adaptive evolution. Additionally, it is expected that the amino acid sites linked to repetitive parallel or convergent variation for certain virulence mutations will show signs of positive selection. Purifying selection is more likely to discard mutations in virus phylogenies that are on shallow branches (i.e. nearer to the tips) due to the fact that they are displayed in a smaller proportion of the population and are thus more prone to be of lower fitness (37).

Phylogenetic modeling of virulence mutations can be performed in two methods, based on how much sufficient and clear data is available. In a “top-down” paradigm, the phylogeny of a virus is determined, mutations are plotted on this phylogeny, and the factors that determine the pathogenicity of mutations remain unclear. “Key branches” include elements like interspecies transmission, geographic expansion, rises in infection rates, spikes in morbidity and/or death, and apparent instances of positive selection. The possible virulence determinants found using this technique may be examined in a laboratory setting (9). The ‘bottom-up’ method, makes use of existing virulence determinants, such as those discovered in an experimental investigation. The phylogenetic position of the putative virulence determinant is then utilized to determine whether it is connected to reciprocal changes that suggest evolutionary trade-offs in order to calculate how it influences virulence evolution. Phylogenomic approaches are increasingly being utilized to identify virulence determinants, but it may also be necessary to formulate generalizations regarding the nature of virulence evolution. A virulence marker intermittently arises on shallow branches and is vulnerable to the rigorous purifying (negative) choice, on the other hand, virulence is not immediately helpful, perhaps because it restricts some other components of overall fitness (10). In such instances, each high-virulence occurrence could be viewed as a separate and temporary evolutionary event. The approach presented here should also be viewed as idealistic, as it works better when a small number of genetic alterations shape pathogenicity independently. However, when there are more complicated interactions between mutations, determining virulence factors may become more difficult (38). Even though RNA viruses are known to have epistasis, nothing is known about how virulence variations behave epistatically. Since RNA viruses have bounded genome sizes, there are likely a handful of virulence determinants, increasing the probability of parallel and convergent evolution, and recombination frequencies are often low within species, hence this strategy may be nicely performed for RNA viruses (39, 40).

## 5 Covid-19

**Figure 1:**
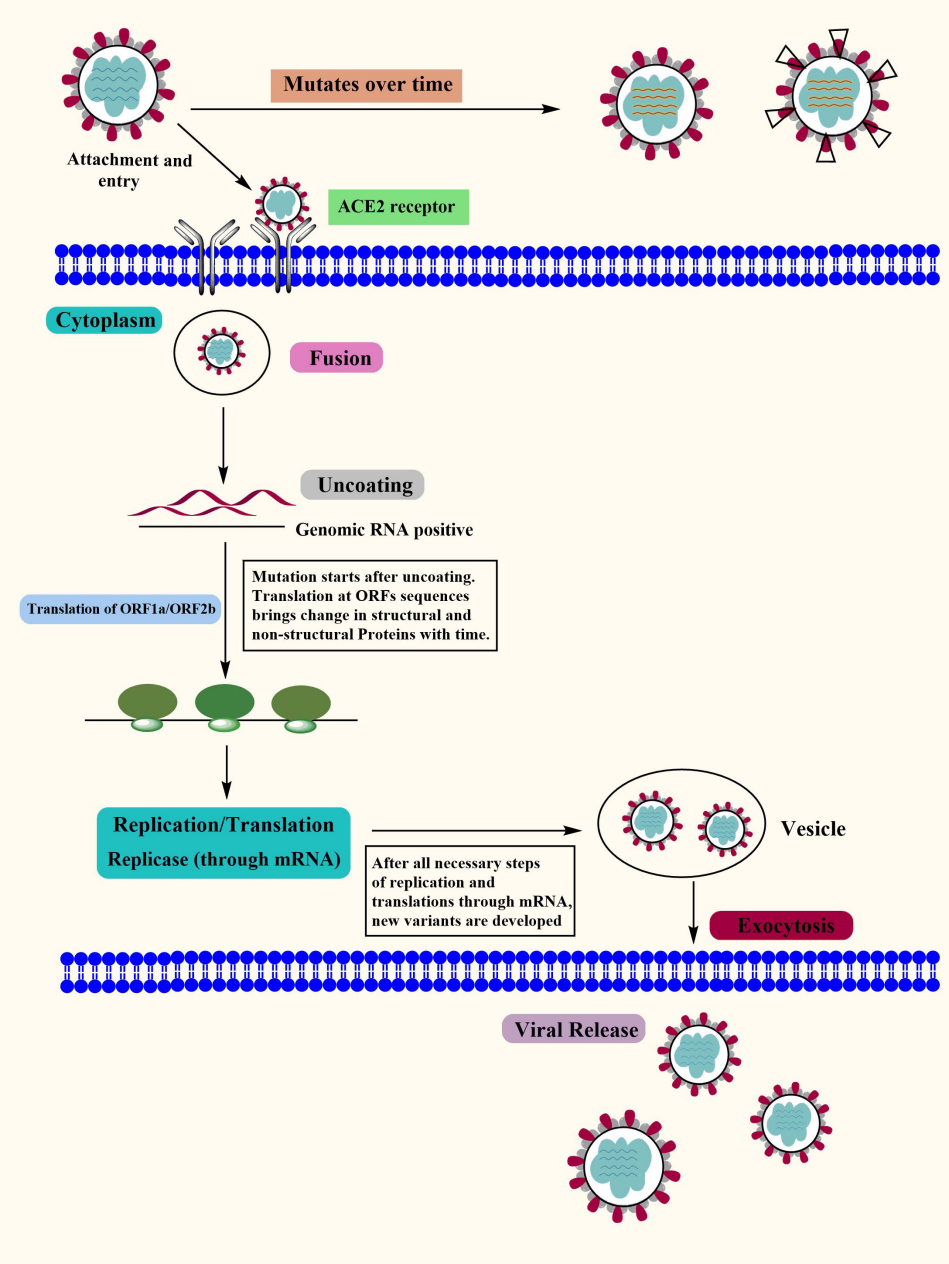
The Covid-19 Life Cycle: The life cycle of Covid-19 in human cells may provide enlightenment for viral transmission and its potential therapeutic targets. Mutations: Mutations in Covid-19 are reported over time as it’s spreading at a very high speed. Although the Covid-19 genome is more stable than SARS-CoV or MERS-CoV, it has a relatively high dynamic mutation rate with respect to other RNA viruses.

Covid-19 is a new highly contagious viral disease that has resulted in thousands of deaths worldwide and has infected millions of people. The coronavirus commonly known as SARS-CoV-2 is the causative agent of Covid-19. Nidovirales is a huge order of viruses and contains different types of virus families including the Coronaviridae, Roniviridae, and Arteriviridae families. Genus coronavirus along with the genus torovirusvirus and bafinivirus belongs to the family Coronaviridae. After Middle East respiratory syndrome coronavirus (MERS) and severe acute respiratory syndrome coronavirus (SARS-CoV), SARS-CoV-2 is the third coronavirus that spreads swiftly across the globe from one person to the next, and various mutations have already been detected (41). Although MERS and SAR-CoV were controlled in a limited time period but the SAR-CoV-2 is still a challenge for healthcare workers in different countries. In some cases, people infected by SARS-CoV-2 develop severe symptoms and even lead to death while in other cases it may cause mild or no symptoms (42). The major symptoms that develop in the affected people include respiratory disorders and fluid accumulation in the lungs resulting in difficulty of breathing. The infected people with or without symptoms are the sources of virus carriers (43, 44). According to observational studies, variables such as age, demographic traits, patient treatment, and other potentially undiscovered factors have a significant impact on clinical outcomes (ranging from asymptotic to mortality) (45–47). A nutritious diet is also acknowledged as an important component in preventing the emergence of severe symptoms by boosting the immune system. Another factor determining the likelihood of Covid-19 development and the prevalence of critical conditions of the illness is the existence of certain underlying medical conditions (e.g., diabetes) in a person (48). The life cycle of SARS-CoV-2 viruses in human cells is shown in **Figure 1**.

## 6 Biology of SARS-CoV-2

**Figure 2:**
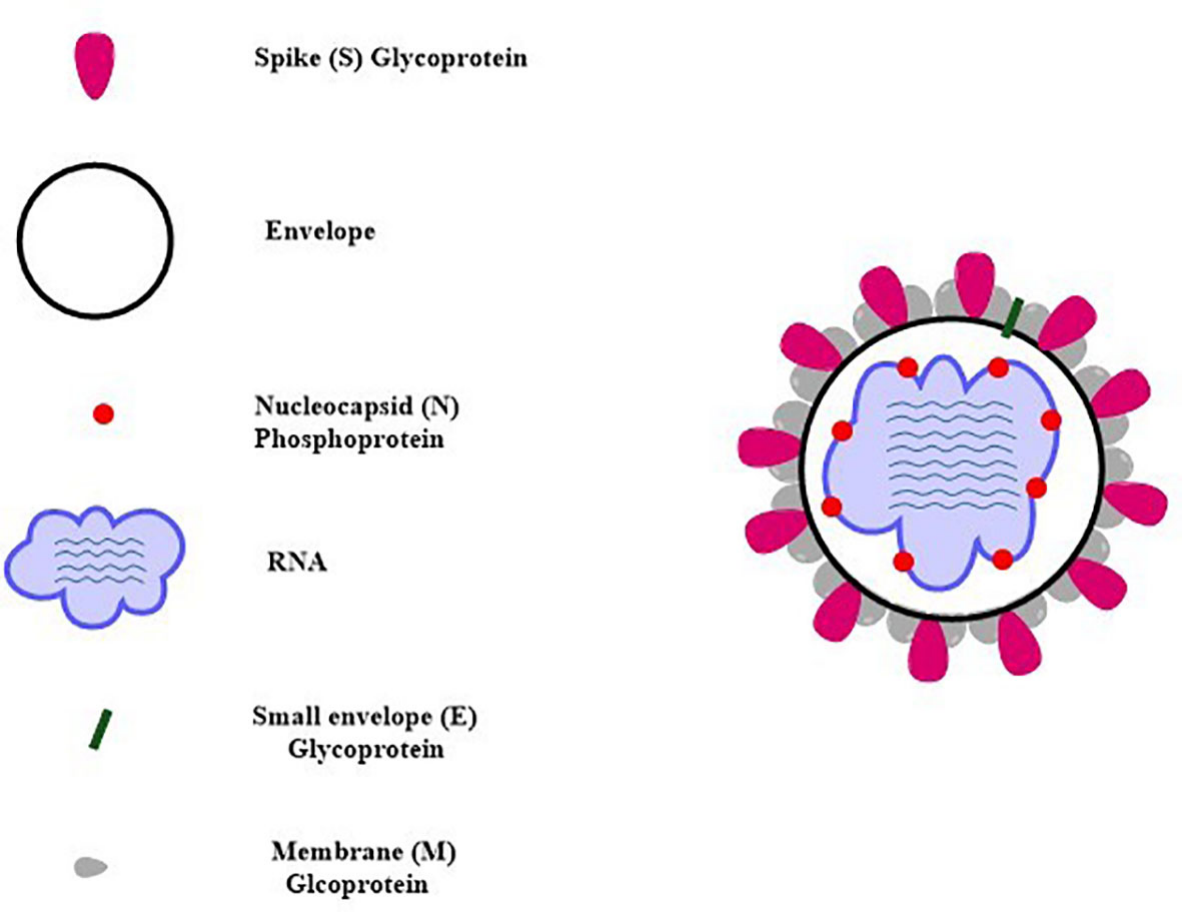
Schematic structure of SARS-CoV-2 with the minimal set of structural proteins.

SARS-CoV-2 virus genome consists of both structural and non-structural proteins encoded in the open reading frame (ORF) sequences. It possesses a single-stranded RNA genome that has a diameter of 60–140 nm and a size of 29.8 to 29.9 kb. SARS-CoV-2 etiology and method of transmission among humans are currently unknown. Since the advent of the SAR-CoV-2 virus, research has been conducted on a wide variety of animal species in an effort to locate potential reservoirs or intermediate hosts for the virus. Its genome is 80% similar to SARS-CoV while 99% similar to that of the bat coronavirus (BAT-CoV) (49). Each of these ORFs has the coding for 17 different structural and non-structural proteins that govern various biological processes throughout the whole life cycle of the virus, from the virus’s ability to survive to its ability to infect other cells (50, 51). The SARS-CoV-2 virus genome begins with the 5′ UTR, which contains a total of 6 to 11 ORF sequences. These genes carry crucial information about virus assembly that has been conserved through many generations. The biological structure of SARS-CoV-2 with the minimal set of structural proteins is shown in **Figure 2**.

### 6.1 Non-structural proteins

The initial step in the virus transition stage is marked by the activation of ORF1ab and ORF1a genomic sequences, which results in the production of two enormous polyproteins called pp1ab and pp1a. The adaptive evolution in ORF1a is shown to contribute to host shifts or immune evasion as a result of selection pressure, and positive selection promotes the evolution of NSPs, shift, and evade the immunological response (52, 53). These two polyproteins are subsequently fragmented into 16 Nsps by two protease enzymes called papain-like proteases, which are present in each of the polypeptides (PLpro) (54). These Nsps are engaged in a wide array of activities, for example, the Nsp3 makes up the coronavirus replication and transcription complex while suppressing the host’s innate immune response. Nsp1 protein plays its role in the inhibition of gene expression of the host cell by binding to the ribosomal subunit thus hindering the entry channel of the mRNA which in turn blocks the formation of mRNA translation assembly (55). Coronavirus NSP 2 genome is homologous to that of the bacterial DNA Topoisomerase I and IV, both of which are necessary for the production of strand RNA. This indicates that NSP 2 could serve as a possible target for the development of pharmaceuticals and vaccinations (56). During replication, 11 cleavage sites between NSP 4 and 16 are processed by 3CL-PRO which is a cystine-like protease found in NSP 5. In addition to that, it possesses a 3-domain structure that has been preserved along with catalytic residues (57–59). RNA replication and pathogenicity are mediated by NSP 9 in association with NSP8 while capping viral mRNA transcripts for effective translation are mediated by the NSP10-NSP16 complex (60–63). The majority of the Nsp proteins that are encoded by ORF1ab participate in the assembly of the replicase-transcriptase complex that can be observed in double-membrane vesicles. In a similar fashion, Nsp8 and Nsp12 are examples of monomeric RdRps that play a role in the replication of SARS-CoV-2. Nsp8, unlike Nsp12, possesses primase capacity, which eliminates the need for primers to begin viral replication. In addition to this, SARS-CoV-2 possesses a unique multimeric RNA polymerase that is composed of the proteins Nsp7 and Nsp8. This RNA polymerase is necessary for the initiation and elongation of the newly created viral genome segment (50, 64). Similarly, exonuclease activity is mediated by NSP 14 in combination with its activator NSP10, while RNA TPase activity is mediated by NSP13 and exoribonuclease activity is mediated by NSP 14, NSP 11 and NSP 15 (65–69). NSP 6 participates in autophagy and produces autophagosomes from the endoplasmic reticulum (70, 71). This study gives a comprehensive assessment of the SARS-CoV-2 polyprotein’s non-structural proteome as well as its unstructured protein domains. This might be useful for comprehending the structural basis of infection, developing structure based therapies and figuring out how SARS-CoV-2 proteins interact with host proteins under varied physiological circumstances (72).

### 6.2 Structural proteins

Spike (S), membrane (M), envelope (E), and nucleocapsid (N) are the four structural protein-encoding genes found at the 3′ end of the SARS-CoV-2 genome which controls various viral processes ranging from virus entry to virus particle production. These proteins are briefly discussed below (54, 73).

#### 6.2.1 S protein

The spike, or S glycoprotein, is a transmembrane protein with a molecular weight of roughly 150 kDa that lies on the outermost layer of the cell membrane. It consists of 1273 amino acid residues split among three subunits (S1, S2, and S2’) that perform distinct roles in cellular adhesion. S protein facilitates viral entrance by interacting with the angiotensin-converting enzyme 2 (ACE2) receptor and facilitating attachment to the plasma membrane of the host cell (44, 74). It protrudes from viral surface in homo-trimers thus determining the spectrum of activity and pathogenicity of the virus (64, 73). The S protein is characterized by a series of structural alterations as it travels through the procedure of entering the host cell (75). It is essential to have an understanding of these conformational changes because dynamic changes in the target protein might alter immunological responses, which is critical for the creation of vaccines (76). Many mutations have been devised in the S protein and there is a high chance that these mutations might alter the antigenicity of the virus (75, 77, 78).

The S2 subunit serves as a fusion protein which plays an important role in the process of the virion and the mammalian cell membrane fusing together. The S2 protein manifests in three different conformational states throughout the fusion process which helps in understanding how these dynamic conformation states coordinate viral entrance into the host cell membrane (76). The last part of the S protein i.e. S2’ functions as a fusion peptide (79). Receptor binding domain (RBD) of the spike protein is the most unstable component of SARS-CoV-2. The 90 amino acid long binding motif of the RBD receptor assists in the virus’s ability to connect to the recipient interface. This receptor binding motif has the slightest conservation, indicating that many mechanisms are likely involved in pathogenesis (72).

In normal conditions, the S proteins of the coronaviruses persist in an inactive state. Upon the entry of the virus into the host cells, the S protein is activated by the host cell proteases and is cleaved into S1 and S2 subunits which in turn activate the membrane fusion domain (44, 80). All kinds of human coronaviruses contain this protein and are responsible for the entrance of viruses into the cells of their hosts. It contains trimeric class I TM glycoprotein that is crucial for the attachment and entry of the virus. During a viral infection, the S protein of SARS-CoV-2 is responsible for facilitating receptor identification as well as cell attachment and fusion (76; 81). Through the RBD region of the S protein, the SARS-CoV-2 virus is able to recognize the ACE2 that is present in the host cell and attach to it. ACE2 is primarily expressed in the alveolar epithelial type II cells, while it is also found in the lung, gut, heart, and kidney (82, 83).

#### 6.2.2 M protein

The shape of the virus membrane is determined by the membrane (M) protein. M proteins are structural proteins having a length of 222 amino acids and work in conjunction with E, N, and S proteins (84). The most prevalent viral proteins in coronaviruses (CoVs) are the M proteins and they play a crucial role in giving the virus its distinctive form. It binds to other structural proteins by operating as a tiny transmembrane protein. During virion assembly development, the M protein helps in the wrapping of the viral genome into a spiral ribonucleocapsid and plays a crucial part in the RNA packing process (64). According to the multiple sequence alignment (MSA) pattern of the M protein, higher sequence conservation is observed in the BAT-CoV, SARS-CoV, and SARS-CoV-2 (72).

#### 6.2.3 N protein

The Nucleocapsid, also known as the N protein, is a major element of SARS-CoV-2 that is attached to the virus’s RNA genome. It also played a key function in wrapping up viral RNA into ribonucleocapsids and is highly conserved among coronaviruses. It participates in several viral genome-related processes, including virus replication, viral genome signaling and the host cell’s response to viral infections. (49, 54). N proteins are therefore regarded as possible therapeutic targets. The RNA-binding domain of the N proteins is around 140 amino acids long and acts as a “bead on a string” to bind viral RNA (85). N protein sequence of SARS-CoV-2 showed high similarity with the SAR-CoV and thus it is speculated that antibodies developed against the former would be likely to detect the latter. The MERS-CoV strain has shown a close similarity, with regions of small sequence differences indicating its dispersion in the process of evolution (72).

#### 6.2.4 E protein

The E protein is a small protein having 75 amino acids and contributes significantly to viral morphogenesis and assembly (86). E Protein is the N-amino terminus of the envelope that is made up of a brief hydrophilic stretch ranging from 7 to 12 amino acids in length. It is followed by the transmembrane domain, which is a big hydrophobic region of 25 amino acids. The C-terminus of this domain is lengthy and hydrophilic, and it makes up the majority of protein. The E protein interacts with the host cell membrane enzyme and is crucial in the virus’s generation and maturation activities (49). E proteins of the SARS-CoV-2 are being explored as a potential therapeutic target.

## 7 Mutations in SARS-CoV-2

SARS-CoV-2 is a single-stranded RNA virus in which mutations occur at a rate of 10^-4^ replacements of bp each year. As discussed above four structural proteins are encoded in the genome of the SARS-CoV-2 i.e. spike protein (S), small protein (E), matrix (M), and nucleocapsid (N) protein (87). The S protein is a type I fusion protein made up of two subunits: S1 is responsible for attaching to receptor, while S2 is responsible for membrane fusion that assembles into trimers on the virion’s surface. SARS-CoV-2 binds with ACE2 receptors to enter the target cells (88). The S protein determines the host’s susceptibility towards infection from the virus and also governs the transmissibility of the virus. Thus all vaccines in development are focused on this protein as it is the primary antigen that triggers the protective immune responses (76, 89).

RNA viruses are believed to mutate more often as compared to DNA viruses. Mutations in the amino acid sequence of the surface protein can have a significant impact on viral function and antibody interactions (90, 91). Study finds that A226V of Chikungunya virus E1 protein and A82V of Ebola virus GP protein were the major contributing factors in viral transmission, pathogenicity and fatality (92, 93). Given the fact that SARS-CoV-2 was recently found in humans, mutations in the gene that codes for the spike (S) protein have been documented several times (94, 95). Glycosylation of viral protein has a significant impact on the viral life cycle which leads to viral mutations. Deactivation or removal of particular glycosylation sites may reduce Env protein interaction with the CD4 receptor, which results in a partial or complete entire infectivity of viral components (96). It has been discovered that alterations in the glycosylation area can have an effect on the cleavage, replication, stability, and antigenicity of HA. Similarly, it has also been discovered that removing certain glycosylation sites from the H5N1 HA protein can have an effect on HA cleavage, replication, stability, and antigenicity (97). Although the S protein has 22 potential N-glycosylation sites and is heavily glycosylated, it is unknown how these sites affect the virus’s ability to infect cells and be neutralized by antibodies (98). Furthermore, changes may emerge with each genome replication cycle. SNPs (single-nucleotide polymorphisms) are used to compare DNA sequences (99). It can be exploited for genetic studies, such as identifying alterations in the coronavirus genome, where additional mutations might potentially exist as a result of a RdRp activating during the replication phase of the genome.

SARS-CoV-2 has dozens of different variations that are reported all over the world. Health specialists closely observe those variants that could pose a problem, often known as “variants of concern.” Alpha variant (B.1.1.7), Beta variant **(**B.1.351**),** Gamma variant (B.1.351), Delta variant (B.1.617.2), and Omicron are the variants of concern among them. Although the majority of SARS-CoV-2 sequence alterations are expected to be harmful and quickly eliminated, a small number of mutations are believed to impact functional characteristics, rate of infection, severity of disease, or contact with the host body’s immune system (100, 101).

### 7.1 Alpha variant (B.1.1.7)

The Alpha variant of SARS-CoV-2, also known as the B.1.1.7, was identified in Kent, England, in September 2020. It possesses the highest risk of transmission of any lineage, with a reproduction rate of 50%–100% (102). When compared to other varieties, it is 40-80% more transmissible, and mortality is expected to be greater than other variants. This variation has a 69/70 deletion and a mutation at nucleotide501 and P681H, which changes the structure of the SARS-CoV-2 spike protein’s receptor-binding domain. At open-reading frame (ORF) 1 a/b, ORF8, spike (S), and N gene areas, it exhibits 23 variations, including 14 amino acids, 8 in the S protein, and three in-frame deletions. These variations have biological consequences and have led to diagnostic problems (103). Phylogenetic investigations revealed that B.1.1.7 has a distinctive accumulation of substitutions and is expanding at a faster rate than other circulating lineages. Alpha variant possesses a limited potential to escape from vaccine-induced immunity as a result vaccines are highly effective toward this variant. It has been reported that Novavax vaccine showed 86% protection for the Alpha variant while PfizerBioNTech vaccine exhibited 92% and 97% effectiveness against asymptomatic and symptomatic infections, respectively. AstraZeneca showed a 94% reduction in hospitalizations (104).

### 7.2 Beta variant (B.1.351)

The first case of the Beta variant of SARS-CoV-2 was reported in South Africa in October 2020. By the end of November 2020, this variant had taken hold throughout Eastern and Western South Africa. This lineage has 23 variations with a total of 17 amino acid alterations in the S gene area, three of which impact the spike protein’s RBD (SK417N, E484K, and N501Y) while some other mutations are in the NTD domains. These three mutations have been reported to possess higher transmissibility as well as conformational changes that could make vaccinations less effective (105, 106). The rest of the mutations are found in viral proteins ORF1a [K1655N], envelope € [P71L], and N [T205I]. Decreased vaccination sensitivity is caused by the mutation E484K in this variant, which arbitrates antibody escape. Several investigations have found that a combination of RBD and NTD alterations in the Beta spike protein has a significant impact on neutralizing activity in vaccine recipients (107, 108). Trials done by Novavax, Janssen, and Astra-Zeneca revealed that the vaccination efficacy was decreased when compared to other versions in which this strain was not prevalent (109). Generally, vaccines have demonstrated high efficacy against the Beta variant of SARS-CoV-2. Pfizer-BioNTech vaccine demonstrated 97.4% effectiveness against severe infections caused by this variant. Janssen’s vaccine exhibited 65%–66% protection against hospitalization while a 91%–95% reduction in mortality rates was notified. AstraZeneca was effective up to 60% toward Beta variant of SARS-CoV-2 (104).

### 7.3 Gamma Variant (P1)

It is also known as the B.1.1.28.1. variant and was recovered on January 2, 2021, from four tourists who landed in Tokyo traveling from Amazonas, Brazil. The sudden rise in the number of hospitalization cases was a primary issue associated with this variation. It was discovered that this variety is 2.2 times more transmissible, resulting in a few cases of contracting the disease in Covid-19 survivors and that infection rates are nearly identical in younger and elderly individuals (110, 111). This variant has 17 non-synonymous mutations in S protein, [S1188L, K1795Q, and E5665D] in ORF1ab, [E92K] in ORF8, and [P80K] in N protein; 1 deletion: [SGF 3675-3677del] in ORF1ab; and four synonymous alterations. P1 variant of Covid-19 acquires a maximum genetic variation of 12 mutations in the S protein (112–114).

The N501Y mutation is common in three forms, but the Beta versions have L18F, K417T, E484K, and D614G mutations. This subset of S variants has significant implications for the attenuation of antibody-mediated protection. Both vaccination and convalescent sera had lower serum neutralization effectiveness against E484K mutation (115–117). Since the receptor binding alterations in B.1.351 and P.1 are similar, immunization efficacy against P.1 should be comparable to B.1.351. Sinovac Biotech has conducted clinical trials in Brazil, which have shown that the CoronoVac vaccine is 50% effective in combating the P.1 variant infection (118, 119).

### 7.4 Delta variant (B.1.617.2)

The B.1.617.2 (Delta) variant is a prepotent SARS-COV-2 variant across the globe which replicates and transmits very quickly resulting in a higher viral burden and severity of infection (120). This variant was first discovered in Maharashtra at the end of 2020 and quickly expanded across India by outnumbering pre-existing lineages such as B.1.617.1 (Kappa) and B.1.1.7 (121). When compared to wild-type Wuhan-1 carrying D614G, B.1.617.2 is six times less susceptible to serum neutralizing antibodies from recovered persons and eight times less susceptible to vaccine-produced antibodies *in vitro*. In the S glycoprotein of this variant, the viral sequences isolated in India exhibited two significant amino acid changes (L452R and E484Q). B.1.617 has three sublineages: B.1.617.1, B.1.617.2, and B.1.617.3, with B.1.617.2 being the most recent variants of concern, the Delta variation (122, 123).

Currently, there is no evidence of whether the transmission routes of B.1.617.2 variant are different from those of the actual SARS-CoV-2. However, it has been noticed that B.1.617.2 transmits more quickly in comparison to the original SARS-CoV-2 (124). Initially, preliminary research suggests that B.1.617.2/Delta cases may have a higher risk of hospitalization than B.1.1.7 cases but the virological characteristics including infectivity and pathogenicity are still unknown (125, 126). There are 17 mutations in the Delta genome, four of which are alarming (127, 128). B.1.617.2 contains seven mutations on its spike proteins including Δ157-158, L452R, T19R, T478K, D950N, P681R, and D614G. In the case of SARS-CoV-2, the junctions of spike proteins contain an amino acid chain composed of proline, arginine, arginine, alanine, and arginine which is termed as furin cleavage site. This site is mutated in B.1.617.2 variant where proline is substituted with arginine (P681R) which decreases the acidity of this sequence. Subsequently, furin host enzymes can efficiently identify and cut the spike proteins on replicated viruses before leaving the host cell, enabling the invasion of more spike proteins to human cells while promoting the fusion between the host cell membrane and viral envelope resulting in severe infection. It has been revealed that approximately 50% of the spike proteins on SARS-CoV-2 interact with human cells while 75% of the spike proteins on B.1.617.2 are available for invasion (124, 128, 129). Furthermore, even though recent reports have indicated that the B.1.617.2/Delta variation is relatively resistant to the neutralizing antibodies (Nabs) induced by vaccination, the mutation(s) responsible for this variant are still unknown (127, 128). Before the outbreak of Delta variant, vaccination potentially reduced the transmission and viral burden in SARS-CoV-2 infected individuals. However, Eyre et al., revealed that vaccination was less efficient to reduce the transmission of Delta variant in comparison to Alpha variant while the effectiveness of vaccination also lessened over time (130).

### 7.5 Omicron variant (B.1.1.529)

During the last phase of 2021, another highly transmissible omicron (B.1.1.529) variant appeared in Africa and quickly became the most prevalent virus across the globe (131). On November 24, 2021, the World Health Organization (WHO) received the first sample of a novel SARS-CoV-2 variant with a significantly altered Spike protein from South Africa, with the first sample collected on November 9, 2021. The Technical Advisory Panel on SARS-CoV-2 virus mutation assessed the variant quickly, and WHO classified Omicron as a variant of concern within 48 hours, allowing for prompt control and prevention. Omicron has been discovered in six continents of the world since its discovery (132–134). Omicron strain is the most distinct variant seen in substantial numbers so far during the outbreak, raising concerns that it may be linked to higher infection rates, lower vaccination efficiency, and a higher risk of reinfection. Omicron version is more communicable than the Delta variant, however, several clinical research studies had showed that omicron infections are less severe than delta infections (133, 135, 136). The Omicron variant employs a unique approach for invading the host and can acquire cell access without any assistance of transmembrane serine protease 2 (TMPRSS2). Omicron enters and reproduces in the host cell by using the endocytic pathway in contrast to Delta variant which uses TMPRSS2. As TMPRS22 is abundantly expressed in alveolar lung cells, hence, lung involvement after host invasion may be absent in the case Omicron variant. Moreover, the fusion potential of Omicron variant is very less in comparison to Delta variant. Hence, the formation of syncytia (a structure generated by the fusion of various cells) becomes quite challenging leading to the emergence of mild clinical symptoms (137). As compared to 4 alarming mutations in Delta, Omicron variant possesses more than 50 mutations, with more than 30 mutations in the spike protein, which enables it to escape from neutralizing potential of antibodies induced by vaccination or earlier infection caused by a non-omicron variant. The omicron lineage is further distributed into subvariants including BA.1, BA.1.1, BA.2, BA.2.12.1, BA.4, and BA.5. Neutralizing antibody titers against BA.5 are reduced up to 3-fold in comparison to the titers against BA.1 and BA.2 (81, 131, 138).

### 7.6 Other SARS-CoV-2 variants

SARS-CoV-2 has developed into several variants since its recognition in November 2019. Different mutations at the genome level have been observed in various parts of the world as new infectious variants emerge. Some of the known variants that have been discovered so far since its emergence are given in **Table 1**.

**Table 1:** Variant lineage and designation of Covid-19.

WHO Nomenclature	Lineage	Designation	Status	Country of detection	Mutation location
Alpha	B.1.1.7	VOC-20DEC-01	VOC	UK	RBD
Beta	B.1.351	VOC-20DEC-02	VOC	South Africa	RBD
Gamma	P.1	VOC-21JAN-02	VOC	Japan ex Brazil	RBD
–	B.1.1.7 with E484K	VOC-21FEB-02	VOC (non-UK)	UK	Spike N-terminal domain
Delta	B.1.617.2	VOC-21APR-02	VOC	India	–
Zeta	P.2	VUI-21JAN-01	VUI	Brazil	–
Eta	B.1.525	VUI-21FEB-03	VUI	UK	–
	B.1.1.318	VUI-21FEB-04	VUI	UK	–
Theta P.3	P.3	VUI-21MAR-02	VUI	Philippines	–
Kappa	B.1.617.1	VUI-21APR-01	VUI	India	E484Q and L452R
–	B.1.617.3	VUI-21APR-03	VUI	India	–
–	AV.1	VUI-21MAY-01	VUI	UK	–
–	C.36.3	VUI-21MAY-02	VUI	Thailand ex Egypt	–
Epsilon	B.1.427/B.1.429	–	Monitoring	–	–
–	B.1.1.7 with S494P	–	Monitoring	–	–
–	A.27	–	Monitoring	–	–
Iota	B.1.526	–	Monitoring	–	E484K and S477N
–	B.1.1.7 with Q677H	–	Monitoring	–	–
–	B.1.620	–	Monitoring	–	–
–	B.1.214.2	–	Monitoring	–	–
–	B.1.1.1 with L452Q and F490S	–	Monitoring	–	–
–	R.1	–	Monitoring	–	–
–	B.1.1.28 with N501T and E484Q	–	Monitoring	Brazil	–
–	B.1.621	–	Monitoring	–	–
–	B.1 with 214insQAS	–	Monitoring	–	–
–	AT.1	–	Monitoring	–	–
Mu	B.1.621, B.1.621.1	–	VBM	–	–
Omicron	B.1.1.529, BA	–	VOC	South Africa	–
–	B.1.618 (Triple mutant variant)	–	Monitoring	India	S Protein
–	A.EU1/ S:A222V	–	Monitoring	Spain	non-terminal domain (NTD)
–	20A.EU2	–	Monitoring	France	S477N, E484K, and N501Y
–	20A/S:439K (S:N439K)	–	Monitoring	Ireland	deletions of amino acids at positions 69 and 70 of S proteins
–	20A/S:98F	–	Monitoring	Belgium, Netherland	S:98F mutation
–	20C/S:80Y	–	Monitoring	–	18 nucleotide mutations
–	20B/S:626S	–	Monitoring	Norway, UK	S:626S mutation
–	20B/S:1122L	–	Monitoring	Sweden, Denmark	S:V1122L mutation
–	N440K	–	Monitoring	India	S protein
–	CAL.20C	–	Monitoring	USA	ORF1a: I4205V, ORF1b: D1183Y, S: S13I; W152C and L452R
–	20C-US or COH.20G/501Y)	–	Monitoring	USA	S protein (Q677H), M protein (A85S) and on the N protein (D377Y)

WHO, World Health Organization; VOC, Variant of Concern; VUI, Monitoring, Variant under Monitoring.

## 8 Pathogenesis of Covid-19

**Figure 3:**
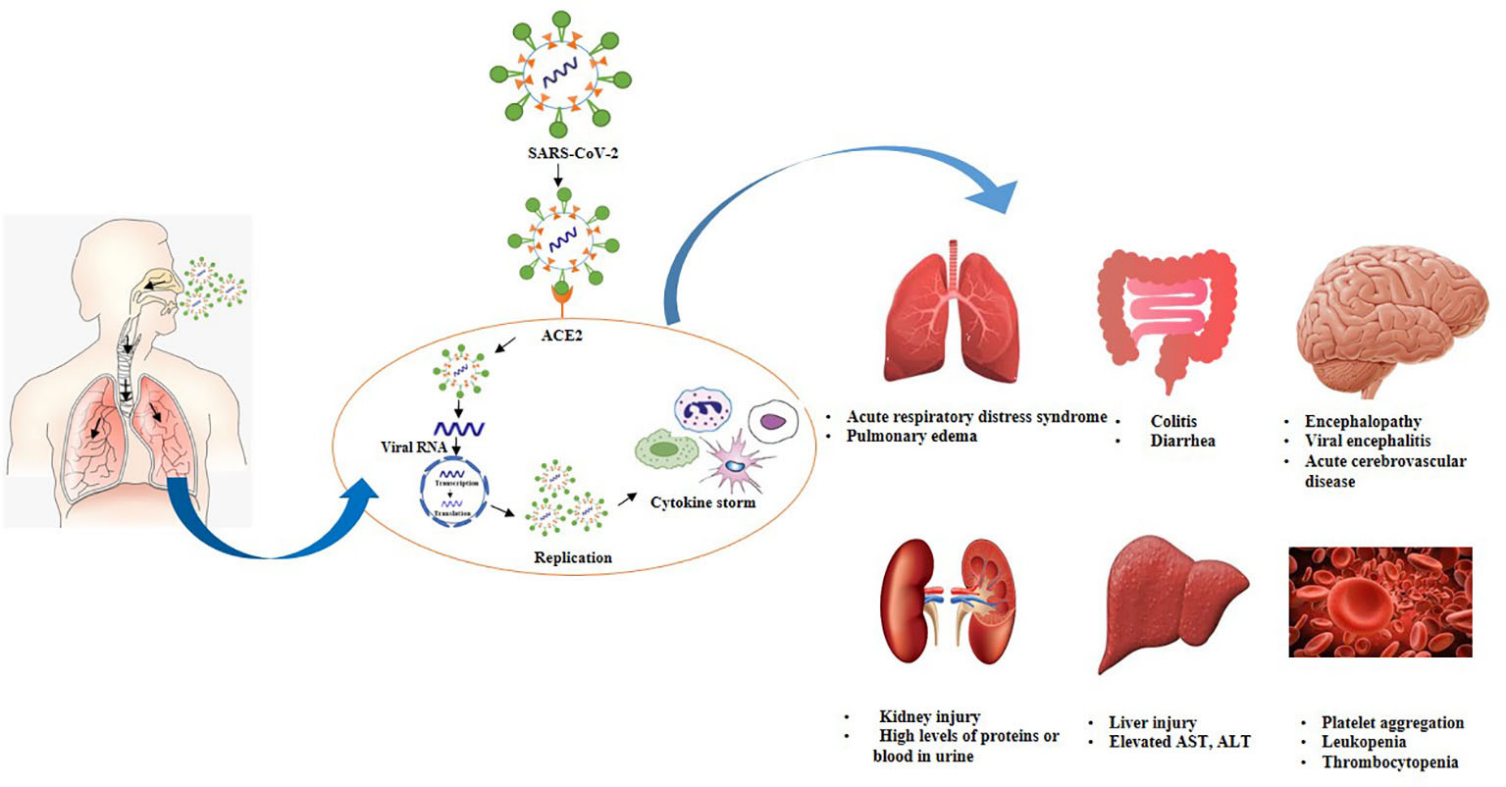
Postulated pathogenesis of SARS-CoV-2 infection. Virus enters through nasal cavity, binds with ACE2 receptors, moves from upper respiratory tract to lower respiratory tract. Virus undergoes replication triggers cytokine syndrome and cause ADRS as well as multiple organ failure.

Individuals infected with SARS-COV-2 exhibit a higher leukocyte count, abnormal respiratory findings, and a higher amount of plasma pro-inflammatory cytokines (139). Currently, the pathogenesis of Covid-19 is not known to a great extent. In-depth studies are still needed to investigate the innate immune response of lung cells. Herein, we have summarized pathogenesis depending on the availability of published information. The pathogenesis of Covid-19 is also described in **Figure 3**. Multiple issues regarding the pathogenesis of Covid-19 are still needed to be explored.

### Phase I. Upper respiratory tract infection

SARS-CoV-2 mainly spreads through respiratory droplets and predominantly replicates in the mucosal epithelium of upper respiratory tract while further multiplication occurs in the lower respiratory tract and gastrointestinal mucosa (140). The inhaled virus initially binds to epithelial cells in the nasal cavity due to the interaction of SARS-COV-2 spike proteins to ACE2 receptors (141, 142). ACE2 receptors are present in the nasal mucosa, bronchus, lung, bladder, stomach, esophagus, heart, kidney, and ileum. However, lung tissues are considered as primary invasion site of SARS-CoV-2 due to a high expression of ACE2 (139, 143–145). Subsequently, cellular protease priming by TMPRSS2 enables S1/S2 subunit breakdown of spike protein while the S2 subunit permits the viral fusion with cell membranes (44). Hence, the entrance of SARS-CoV-2 into cells is mainly facilitated by the direct membrane fusion between the virus and plasma membrane. Moreover, clathrin-dependent and -independent endocytosis also promotes viral invasion (143, 146). After virus infiltrates into the cells, viral RNA genome liberates into the cytoplasm which is translated into two polyproteins and structural proteins, leading to the replication of viral genome (147). Freshly formed glycoproteins penetrate the membrane of the endoplasmic reticulum or Golgi, and the nucleocapsid generates by the amalgamation of genomic RNA and nucleocapsid protein. Subsequently, viral particles germinate into the endoplasmic reticulum-Golgi intermediate compartment (ERGIC). Ultimately, vesicles holding the virus particles merge with plasma membranes to liberate the virus (148, 149). It is believed that viral replication mainly takes place in the mucosal epithelium of the upper respiratory tract (150). During this phase, viral burden is usually low which triggers a limited innate immune response however, virus can be detected by nasal swabs (145). Usually, viral infection is eradicated at this phase by the secretion of type I or type III interferon, and generation of B and T cell responses. Although in a few cases, virus can disseminate to the lower respiratory tract (151).

### Phase II. Lower respiratory tract infection

SARS-CoV-2 can move into the lungs through the inhalation of virus particles from the upper respiratory tract. Virus further propagates either by directly infecting the cells in the lower respiratory tract or by invading the airway cells across the tracheobronchial tree. It has been revealed that ciliated cells in the lower respiratory tract serve as primary target sites for SARS-CoV-2 (151, 152). Moreover, in a few cases, the virus may interact with club cells as well. Viral invasion in ciliated cells promotes the loss of ciliation of bronchial epithelial cells which can impede the upward movement of mucus in airways. This phenomenon promotes the spread of the virus into the alveoli where it interacts with AT2 cells which express ACE2 receptors. Subsequently, viral interaction with AT2 cells triggers type I and type III interferon responses. In short, interferon release, inflammation, apoptosis, the inability of surfactant production, and AT2 identity are the manifestations of virus replication (151, 153). Viruses, as well as early markers of the innate immune response, can be detected in nasal swabs or sputum. During this stage, Covid-19 morbidity is clinically manifested (145).

### Phase III. Cytokine storm

It is a well-known fact that cytokines play a vital role in the development of viral infection. A robust and organized innate immune response serves as the first line of defense against viral infection. However, unregulated and extravagant immune responses can cause immune damage to the human body. Various shreds of evidence from severe conditions of Covid-19 refer that proinflammatory responses are involved in the progression of this disease. The release of interferon (IFN)-I or IFN-α/β is a primary immune defense response against viral invasion, hence IFN-I is the main agent that copes with the early stages of viral infection. It has been revealed that delayed liberation of IFNs during the initial phases of SARS-CoV invasion impedes the body’s antiviral response (154). As the virus reaches the lungs, immune system sends various immune cells including pro-inflammatory cytokines as well as chemokines to the lung tissue to combat the virus. Although, immune cells attack indiscriminately as these are unable to locate the virus and promote the recruitment of more immune cells to combat the virus hence, generating a cytokine storm (139, 155).

Cytokine storm is a robust immune response that triggers the release of cytokines into the systemic circulation (140, 156). During the initial phases of SARS-CoV-2 invasion, cytokines and chemokines are released in a delayed manner inside the respiratory epithelial cells, dendritic cells (DCs), and macrophages which impedes the body’s antiviral response. Subsequently, the cells release less amount of antiviral factors i.e., IFNs, and a huge quantity of proinflammatory cytokines including tumor necrosis factor (TNF), interleukin IL-6, IL-1β, chemokines, chemokine ligand CCL-2, CCL-3 and CCL-5. Furthermore, cytokines and chemokines attract various inflammatory cells including monocytes and neutrophils leading to extravagant permeation of the inflammatory cells into lung tissue and promotes lung injury (154, 157, 158). Once a cytokine storm is triggered, the immune system is unable to kill the virus however, it also kills a wide range of normal lung cells and affects the functioning of the lungs (139).

On the other hand, suppression of pulmonary ACE2 function causes impairment of renin-angiotensin system (RAS), promotes inflammation and enhance vascular leakage and ultimately leads to acute lung injury (140). Elevated levels of cytokines and chemokines were observed in severe cases including IL-7, IL-8, IL-9, IL-10, IL-1RA, and IL1-β (159). Different markers of hyperinflammation including IL-6, IL-8, IL-2R, IL-10, inducible protein (IP10), monocyte chemoattractant protein-1 (MCP1), TNF-alpha are remarkably higher in individuals who died as compared to survivors (160). The Elevated level of IL-6 is an indicator of severe Covid-19 cases and a major component of cytokine storm (161).

### Phase IV. Acute respiratory distress syndrome (ARDS)

It has been noticed that in severe Covid-19 cases presence of cytokine storm ultimately causes acute respiratory distress syndrome (ARDS) (162). ARDS is a life-threatening state that circumvents the entrance of oxygen into the lungs and circulation resulting in mortality and acute lung injury (140). Inflammatory action triggered by the release of excessive cytokines cause alveolar cell damage and necrosis (163). Enhanced endothelial and epithelial permeation facilitates the development of alveolar edema. Subsequently, alveolar barrier and osmotic gradient which promotes the clearance of alveolar fluid are disturbed. Necrosis and edema mediate a more robust immune response. Moreover, pulmonary edema prevents gas exchange and reduces carbon dioxide removal resulting in hypoxemia and acute respiratory failure (164). Approximately 20% of the infected patients developed hypoxia and ARDS leading to multiple organ failures (165). In fatal cases of Covid-19, patients encounter severe respiratory distress and need mechanical ventilation (140). Moreover, various studies have explained the role of genetic susceptibility in the development of ARDS. Up to 40 genes including ACE2, TNF, and vascular endothelial growth factor (VEGF) are attributable to the development of ARDS (140).

### Phase V. Effects on other organs

ACE2 receptors are also present in other organs such as the central nervous system, cardiovascular system, gastrointestinal tract, and kidneys, hence all these organs are susceptible to viral attack. Cytokine storm is not only attributable to lung tissue damage and ARDS but also causes tissue damage in other organs which results in multiple organ failures (159). Viral invasion triggers the systemic inflammatory response which disturbs the balance between procoagulant and anticoagulant homeostatic mechanisms (144). It has been noticed that Covid-19 patients have the potential risk of disseminated intravascular coagulation (166 84). SARS-COV-2 adhesion to ACE2 activates renin-angiotensin-aldosterone system (RAAS) which carries the risk of platelet aggregation and causes pulmonary embolism as well as fibrosis (167, 168). In severe cases, myocardial ACE2 pathways are extremely downregulated which causes myocardial injury and leads to death (169, 170). Damage of endothelial cells mediated by viral infection leads to excessive thrombin formation and inhibition of fibrinolysis as a result Covid-19 patients may exhibit hypercoagulability. Moreover, the immobility of patients or inpatient treatment for prolonged time intervals enhances the risk of venous thromboembolism in Covid-19 patients (140, 167, 171).

SARS-COV-2 infection also affects the gastrointestinal tract which is enriched with ACE2 receptors. Diarrhea has been reported in approximately 20% of Covid-19 patients however, the mechanism is not completely elucidated. Viral infection changes intestinal permeability and promotes enterocyte malabsorption (172). ACE2 is responsible for the uptake of dietary amino acids and triggers the homeostasis of gut microbiota. It has been revealed that SARS-CoV-2 can induce ACE2 mutations and promote susceptibility to colitis, and diarrhea (172, 173).

SARS-COV-2 may induce liver damage but the mechanism is not fully known. It is proposed that liver enzymes may be elevated as a result of a cytokine storm. SARS-CoV-2 can interact with endothelial cells in the bile duct and triggers inflammatory damage to the liver. Furthermore, virus can interact with kidneys either directly or induce kidney damage through systemic effects including low blood pressure (165, 174, 175).

Some Covid-19 patients also encounter encephalitis, stroke, and epilepsy which depicts that virus also affects nerve cells. Patients also lose their sense of smell as olfactory nerve endings are affected. SARS-CoV-2 can trigger neurological disorders by penetrating the nervous system either through olfactory nerve or neuronal pathways (144, 176). It is assumed that pathological changes affecting the vital organs in Covid-19 patients can be caused either directly by the cytopathic effect or indirectly due to the harmful immune responses mediated by SARS-CoV-2. However, the estimation of the comparative significance of each of these factors demands further investigation (86).

## 9 Vaccine strategies against Covid-19

Serious and swift efforts have been made in the development and production of vaccines against Covid-19. Many different groups from all over the world have made laborious attempts to produce an effective vaccine for Covid-19. Some of the efforts so far are successful while some did not get the required results. Various strategies are being used for the development of vaccines against Covid-19. The main purpose of these strategies is to develop immunity against the given virus and to protect precious lives with minimum or no side effects (119, 177, 178). Different vaccines target either whole virus or any fragment of its body depending upon its method of development. Storage conditions and socioeconomic factors are also considered important for developing vaccines so that all people irrespective of color, country and religion can get the vaccine. Different strategies for the development of vaccines against viruses are discussed below while the details of all vaccines are given in **Table 2**.

**Table 2:** Vaccines approved for full use or in limited use against Covid-19.

S.No:	Vaccine name	Country	Vaccine type	Approved
1	Vaxine/CinnaGen Co.SpikoGen	Australia	Protein Subunit	Approved in 1 country
2	Zydus CadilaZyCoV-D	India	DNA	Approved in 1 country
3	Anhui Zhifei LongcomZF2001	China	Protein Subunit	Approved in 3 countries
4	Biological E LimitedCorbevax	India	Protein Subunit	Approved in 1 country
5	CanSinoConvidecia	China	Non-replicating Viral Vector	Approved in 10 countries
6	Center for Genetic Engineering and Biotechnology (CIGB)Abdala	Cuba	Protein Subunit	Approved in 6 countries
7	Chumakov CenterKoviVac	Russia	Inactivated	Approved in 3 countries
8	FBRIAurora-CoV	Russia	Protein Subunit	Approved in 1 country
9	FBRIEpiVacCorona	Russia	Protein Subunit	Approved in 4 countries
10	GamaleyaSputnik Light	Russia	Non-replicating Viral Vector	Approved in 26 countries
11	GamaleyaSputnik V	Russia	Non-replicating Viral Vector	Approved in 74 countries
12	Health Institutes of TurkeyTurkovac	Turkey	Inactivated	Approved in 1 country
13	Instituto Finlay de Vacunas CubaSoberana 02	Cuba	Protein Subunit	Approved in 4 countries
14	Instituto Finlay de Vacunas CubaSoberana Plus	Cuba	Protein Subunit	Approved in 1 country
15	Janssen (Johnson & Johnson)Ad26.COV2.S	USA	Non-replicating Viral Vector	Approved in 106 countries
16	Kazakhstan RIBSPQazVac	Kazakhstan	Inactivated	Approved in 2 countries
17	MedigenMVC-COV1901	Taiwan	Protein Subunit	Approved in 2 countries
18	Minhai Biotechnology CoKCONVAC	China	Inactivated	Approved in 2 countries
19	ModernaSpikevax	USA	RNA	Approved in 85 countries
20	National Vaccine and Serum InstituteRecombinant SARS-CoV-2 Vaccine (CHO Cell)	China	Protein Subunit	Approved in 1 country
21	NovavaxNuvaxovid	USA	Protein Subunit	Approved in 36 countries
22	Organization of Defensive Innovation and ResearchFAKHRAVAC (MIVAC)	Iran	Inactivated	Approved in 1 country
23	Oxford/AstraZenecaVaxzevria	UK	Non-replicating Viral Vector	Approved in 138 countries
24	Pfizer/BioNTechComirnaty	USA/Germany	RNA	Approved in 137 countries
25	Razi Vaccine and Serum Research InstituteRazi Cov Pars	Iran	Protein Subunit	Approved in 1 country
26	Serum Institute of IndiaCovishield (Oxford/ AstraZeneca formulation)	India in Collaboration	Non-replicating Viral Vector	Approved in 47 countries
27	Serum Institute of IndiaCOVOVAX (Novavax formulation)	India	Protein Subunit	Approved in 3 countries
28	Shifa Pharmed Industrial CoCOVIran Barekat	Iran	Inactivated	Approved in 1 country
29	Sinopharm (Beijing)Covilo	China	Inactivated	Approved in 89 countries
30	Sinopharm (Wuhan)Inactivated (Vero Cells)	China	Inactivated	Approved in 2 countries
31	SinovacCoronaVac	China	Inactivated	Approved in 53 countries
32	TakedaTAK-919 (Moderna formulation)	Japan	RNA	Approved in 1 country
33	Bharat BiotechCovaxin	India	Inactivated	Approved in 13 countries

It is necessary to update the thorough study of Covid-19 vaccines, specifically during the Covid-19 pandemic produced by SARS-CoV-2 Delta and Omicron variants (179). The majority of research has been done to assess how well Covid-19 vaccines, particularly mRNA vaccines, work in high-income nations. They used information from trustworthy databases that were connected together, but low-and middle-income nations frequently lack access to such databases due to which, no similar studies were conducted in these nations (180). One of the top worldwide hazards to public health has been highlighted as vaccine hesitancy, which has been a widespread issue in the civilized world for years. There is still hesitation to receive Covid-19 immunization despite the fact that the vaccine being available for more than a year (181). The establishment of faith in medical professionals and manufacturers of vaccines needs to be a priority. In addition to this, the transmission of trustworthy information on the Covid-19 vaccine through the internet and other platforms is also very essential (182).

## Conclusion

The likelihood of mutations increases as the virus spreads. The genomes of SARS-CoV-2 isolates from various countries across the world show a large number of mutations displayed in different viral proteins. A wide range of point mutations and deletions are constantly evolving genetically in the spike protein which has resulted in the discovery of a large number of mutations in SARS-CoV-2. The new mutations not only increase transmissibility, morbidity, and mortality, but they can also elude identification by diagnostic techniques. These variants have a lower susceptibility to treatment, such as antivirals, monoclonal antibodies, and convalescent plasma, and have the potential to reinfect the vaccinated people. Individuals with compromised immune systems are at an increased risk to develop severe inflammatory syndrome. To tackle the Covid-19 threat, it is necessary to identify short-term preventive options as well as long-term immunizations. Despite substantial advancements and positive results from vaccine candidate trials, various obstacles are still present including the delivery of millions of doses to the global population in order to stop the spread of the virus and minimize various mutations. The emergence of SARS-CoV-2 variations is linked to antibody escape from viral spike epitopes, which poses a major risk of re-infection and endangers the effectiveness of all vaccines. It’s also worth noting that new variants may further increase the disease’s severity, transmission, and complexity, therefore it is critical to limit the spread of the virus by following all preventive measures. Effective therapeutic interventions are likely to come from consistent research efforts and a complete understanding of Covid-19 pathogenesis. Despite the rapid spread of virus around the globe, the world has witnessed tremendous scientific cooperation and collaboration, which will undoubtedly serve as a model for future pandemic responses.

## Author contributions

Author contributions. Conceptualization. MK, AN, JS, MAF and WJ Writing Original manuscript. MK, AN, MIA and HA-F Graphical work. MK, AN, IA and MAF Review and editing. MIA, HA-F, JS, MAF and WJ. All authors contributed to the article and approved the submitted version.

## Conflict of interest

The authors declare that the research was conducted in the absence of any commercial or financial relationships that could be construed as a potential conflict of interest.

## Publisher’s note

All claims expressed in this article are solely those of the authors and do not necessarily represent those of their affiliated organizations, or those of the publisher, the editors and the reviewers. Any product that may be evaluated in this article, or claim that may be made by its manufacturer, is not guaranteed or endorsed by the publisher.
